# Failures in thymus medulla regeneration during immune recovery cause tolerance loss and prime recipients for auto-GVHD

**DOI:** 10.1084/jem.20211239

**Published:** 2021-12-15

**Authors:** Abdullah S. Alawam, Emilie J. Cosway, Kieran D. James, Beth Lucas, Andrea Bacon, Sonia M. Parnell, Andrea J. White, William E. Jenkinson, Graham Anderson

**Affiliations:** Institute for Immunology and Immunotherapy, College of Medical and Dental Sciences, Medical School, University of Birmingham, Birmingham, UK

## Abstract

Bone marrow transplantation (BMT) is a widely used therapy for blood cancers and primary immunodeficiency. Following transplant, the thymus plays a key role in immune reconstitution by generating a naive αβT cell pool from transplant-derived progenitors. While donor-derived thymopoiesis during the early post-transplant period is well studied, the ability of the thymus to synchronize T cell development with essential tolerance mechanisms is poorly understood. Using a syngeneic mouse transplant model, we analyzed T cell recovery alongside the regeneration and function of intrathymic microenvironments. We report a specific and prolonged failure in the post-transplant recovery of medullary thymic epithelial cells (mTECs). This manifests as loss of medulla-dependent tolerance mechanisms, including failures in Foxp3^+^ regulatory T cell development and formation of the intrathymic dendritic cell pool. In addition, defective negative selection enables escape of self-reactive conventional αβT cells that promote autoimmunity. Collectively, we show that post-transplant T cell recovery involves an uncoupling of thymopoiesis from thymic tolerance, which results in autoimmune reconstitution caused by failures in thymic medulla regeneration.

## Teaser

The thymus ensures immune tolerance by synchronizing thymocyte development and selection. Alawam et al. show that a functional uncoupling of these events occurs after bone marrow transplantation, which results in post-transplant tolerance loss and autoimmunity caused by selective failures in thymus medulla regeneration.

## Graphical Abstract

**Figure:**
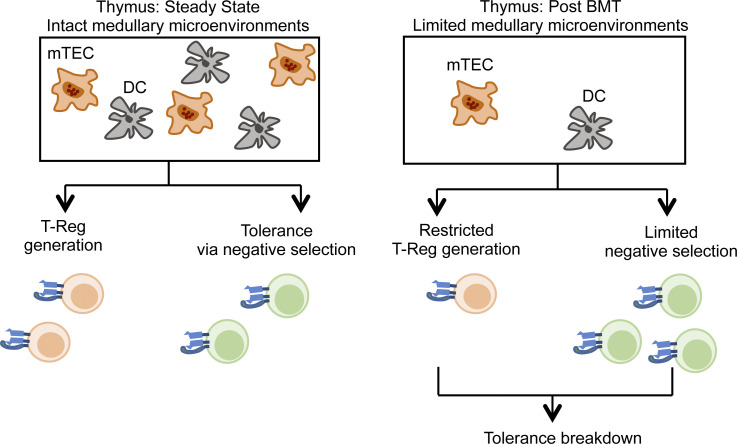

## Introduction

The thymus is critical for the development and function of a self-tolerant immune system. At steady state, ongoing thymopoiesis ensures the continued appearance of double-positive CD4^+^CD8^+^ and single-positive (SP) CD4^+^ and CD8^+^ thymocytes from bone marrow (BM)–derived progenitors (Krueger et al., 2017; Takada et al., 2014). In addition, the thymus also ensures MHC-reactive αβT cells generated by positive selection on cortical thymic epithelial cells (cTECs) are rendered tolerant to self-antigens. Such tolerance events prevent autoimmunity and are primarily controlled by the thymus medulla and interactions with medullary TECs (mTECs) and dendritic cells (DCs; Cosway et al., 2017; Hogquist et al., 2005). For example, negative selection eliminates SP thymocytes that express self-reactive αβTCRs, ensuring thymic export is biased toward self-tolerant αβT cells (Daley et al., 2017; Gavanescu et al., 2007). Additionally, mTEC and DC control lineage divergence in CD4^+^ SP thymocytes to generate Foxp3^+^ regulatory T cells (Tregs) that restrain autoreactivity in peripheral tissues (Breed et al., 2018; Cowan et al., 2013; Perry et al., 2014). Thus, under homeostatic conditions the medulla enables the synchronized production and tolerization of αβT cells, which maintains the correct balance of tolerance and immunity.

The thymus is also important in clinical therapies for immune disorders. For example, during treatment of hematological cancers by host (autologous) or donor (allogeneic) BM transplantation (BMT), the thymus must support de novo production of naive αβT cells from transfused progenitors in order to achieve long-lasting immune reconstitution in post-transplant patients (Awong et al., 2013; Hakim et al., 2005; Singh et al., 2020). Importantly, allogenic BMT can result in graft-versus-host disease (GVHD), a damaging condition that is caused by donor T cell reactivity against host tissues (Arber et al., 2013; Barton-Burke et al., 2008; Cooke et al., 2017). Interestingly, several studies have also reported a condition, sometimes termed "auto-GVHD" or "autoaggression syndrome" (Sprent et al., 1992; Kline et al., 2008; El-Jurdi et al., 2017), which can occur following transplantation of autologous BMT. Importantly, the autoimmune symptoms that occur following autologous BMT cannot be explained by alloreactivity. However, despite its potential relevance to both therapeutic immune reconstitution and immune system function, the underlying mechanisms responsible for auto-GVHD are poorly understood. Finally, while donor thymopoiesis in mouse models of BMT is often used as an indicator of successful thymus recovery after BMT (Chaudhry et al., 2017; Muraro et al., 2005; Tormo et al., 2017), whether this occurs in parallel with the reestablishment of central tolerance mechanisms that prevent autoimmunity is unclear.

Here, we examine the ability of the thymus to support T cell recovery alongside synchronized reestablishment of immune tolerance. Using a syngeneic mouse model of immune ablation and BMT, we report that mTECs but not cTECs show a prolonged failure during initial stages of post-transplant immune reconstitution. This imbalance results in the ineffective development of medulla-dependent Foxp3^+^ Tregs and a failure to regenerate an intrathymic DC pool. Importantly, while medullary abnormalities do not prevent conventional thymocyte development, they lead to failures in negative selection, resulting in escape of self-reactive αβT cells that promote autoimmunity. Collectively, we show that the two key functions of the thymus, T cell development and T cell tolerance, become separated following therapeutic interventions that include BMT. This identifies the thymus medulla as a site of critical importance in the reestablishment of a safe and effective immune system during immune reconstitution.

## Results and discussion

### Sustained failures in thymus medulla regeneration follow immune reconstitution

**Figure S1.**
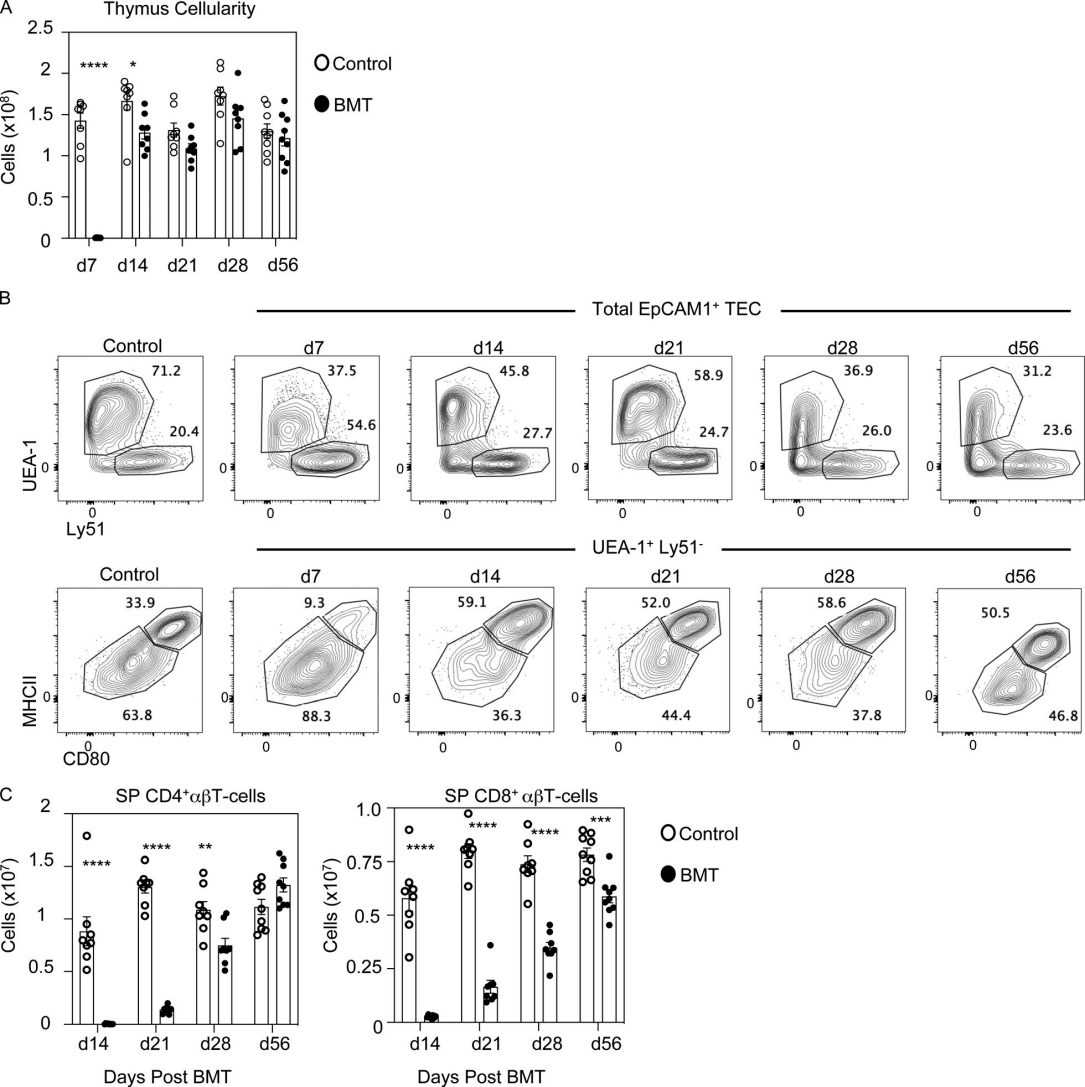
**Thymus cellularity, TEC subset definition, and peripheral T cells in BMT mice.** **(A–C) **Total thymus cellularity is shown across all time points analyzed after BMT (A), cTEC/mTEC gating (B), and number of CD4^+^ and CD8^+^ αβT cells in the spleen (C) of age-matched control and BMT mice (donor-derived CD45.1^+^ cells for the latter) at the indicated time points after transplant (minimum of eight mice from at least three separate experiments). d, day. Error bars indicate SEM. *, P < 0.05; **, P < 0.01; ***, P < 0.001; ****, P < 0.0001.

**Figure 1.**
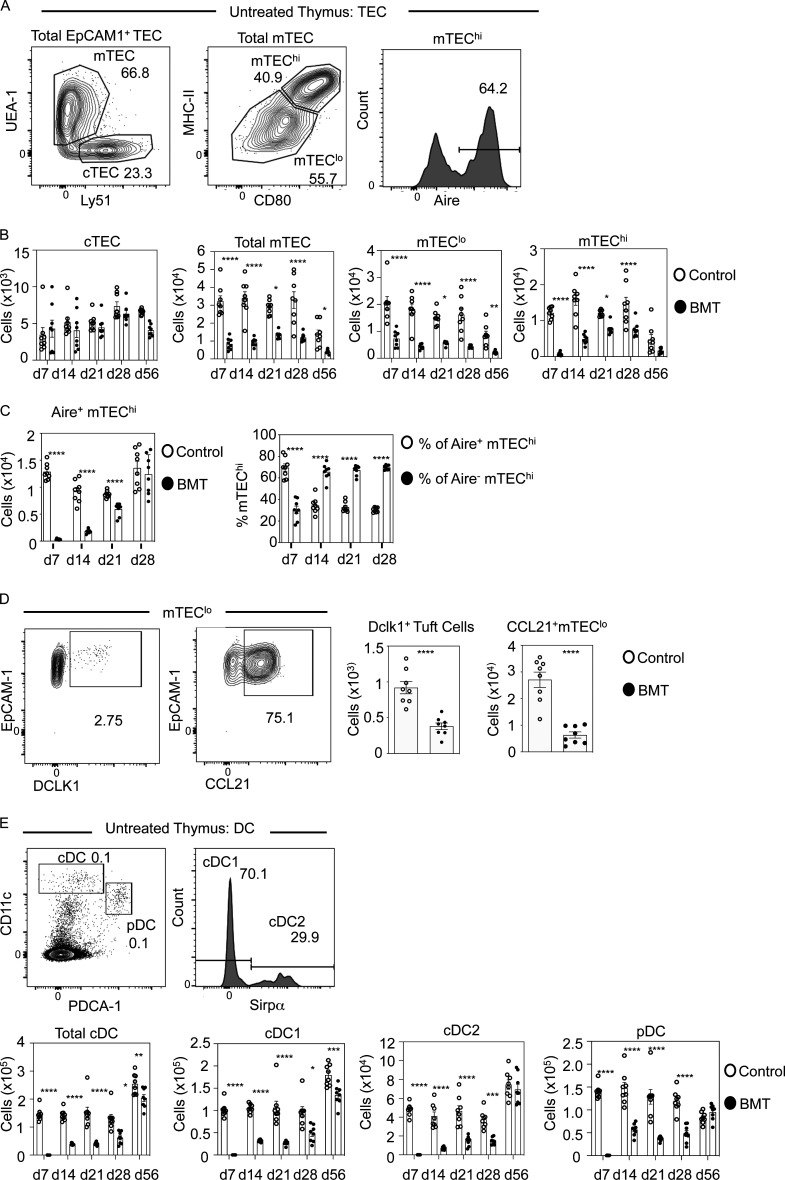
**Selective and sustained failures in thymus medulla regeneration during BMT-mediated immune reconstitution.** **(A)** Gating strategy for TEC subsets in control and post-transplant thymus tissue. **(B)** Quantitation of TEC subsets in control (white dots) and BMT (black dots) mice. Controls are age-matched cohorts of unmanipulated mice taken at each time point alongside transplanted mice. Data represent three experiments, eight mice for each time point. d, day. **(C)** Quantitation of number of Aire^+^ mTEC^hi^ cells in control (white dots) and after BMT (black dots) and proportions of Aire^+^ or Aire^−^ cells within mTEC^hi^. **(D)** Representative FACS plots of DCLK1 or CCL21 expressing mTEC^lo^ with quantitation of these in control (white dots) and BMT mice (black dots); *n* = 8 across two independent experiments. **(E)** Gating strategy to detect thymic PDCA-1^+^ plasmacytoid DC, Sirpα^−^ cDC1, and Sirpα^+^ cDC2 in control and post-transplant mice with quantitation of thymic DCs in control (white bars) and BMT (black bars) mice. Data from three separate experiments, *n* = 8 each time point. Error bars indicate SEM. *, P < 0.05; **, P < 0.01; ***, P < 0.001; ****, P < 0.0001.

**Figure S2.**
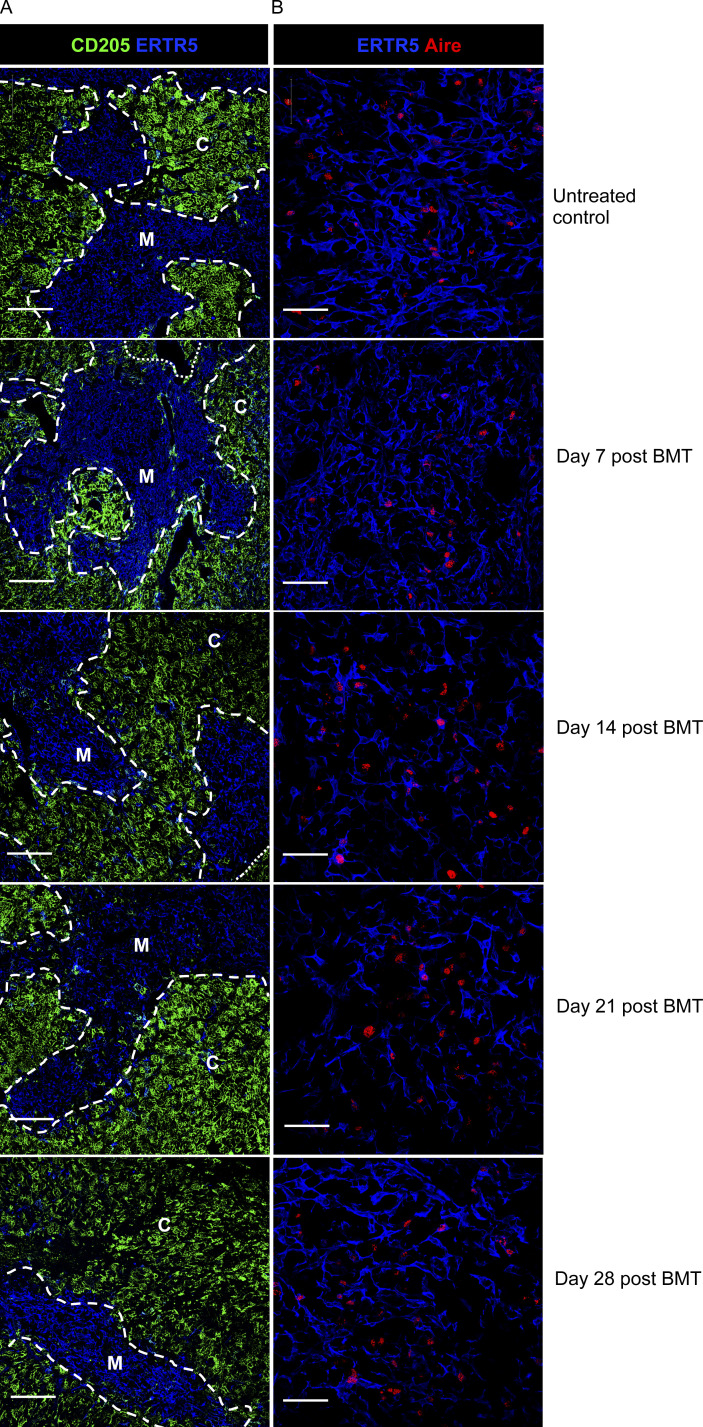
**Thymus microenvironments in BMT mice.** **(A and B) **Confocal analysis of frozen thymus sections from untreated and post-BMT mice at the indicated time points are shown, labeled with CD205 and ERTR5 to identify cortex and medulla (A) and ERTR5 and Aire to detect Aire^+^ mTECs (B). Dashed/dotted lines denote corticomedullary junction. Scale bars denote 200 μm (A) and 50 μm (B). C, cortex; M, medulla.

To examine how intrathymic microenvironments relate to thymus function during immune reconstitution after BMT, we generated syngeneic BM chimeras (Chung et al., 2001; Dudakov et al., 2012; Lucas et al., 2016), where female adult WT CD45.2^+^ C57BL/6 mice were subjected to split-dose (2 × 500 rad), total-body lethal irradiation, then injected with T cell–depleted BM from female adult WT CD45.1^+^ donors. We studied post-transplant thymus function in a 56-d period where initial waves of donor thymopoiesis reestablish thymus cellularity and generate donor-derived peripheral T cells (Fig. S1, A and C; Dudakov et al., 2012; Lucas et al., 2016). At all time points studied, unmanipulated age/gender/strain–matched mice were used as controls. At harvest, thymuses were digested, and EpCAM1^+^ TECs were subdivided into UEA1^−^Ly51^+^ cTECs and UEA1^+^Ly51^−^ mTECs (Fig. 1 A and Fig. S1 B), which were further subdivided into MHCII^lo^CD80^lo^ mTEC^lo^ and MHCII^hi^CD80^hi^ mTEC^hi^, the latter containing Aire^+^ cells (Fig. 1 A). Interestingly, no significant differences in cTECs were observed in BM chimeras and control mice during the 56-d time course (Fig. 1 B). In contrast, mTECs were significantly reduced at 7 d, which remained detectable until at least 56d after BMT, and included reduced mTEC^lo^ and mTEC^hi^ (Fig. 1 B). When we analyzed Aire^+^ mTEC^hi^, their numbers were significantly decreased at 7 d, 14 d, and 21 d but returned to control numbers by 28 d (Fig. 1 C). This correlated with the progressive paucity, but then dominance, of the proportion of Aire^+^ cells within total mTEC^hi^ across a 28-d time course (Fig. 1 C). Whether this indicates that the regenerating thymus supports preferential maturation of Aire^+^ mTECs and/or a block at the Aire^+^ stage that causes a reduction in post-Aire stages (Bornstein et al., 2018; Miller et al., 2018; Yano et al., 2008) is not clear. To examine this, we examined mTEC heterogeneity by analyzing frequencies of thymic tuft cells, representing a post-Aire stage in mTEC development (Bornstein et al., 2018; Miller et al., 2018) and mTEC^lo^ expressing CCL21 (Lkhagvasuren et al., 2013). Both tuft cells and CCL21^+^mTEC^lo^ were significantly reduced at 28 d after transplant (Fig. 1 D). While reasons for differential recovery of individual mTEC^hi^ and mTEC^lo^ that occurs after BMT are unknown, our findings indicate the post-transplant thymus contains a deficiency in the mTEC but not the cTEC compartment. Despite the absence of donor double-positive CD4^+^CD8^+^ and SP CD4^+^ and CD8^+^ thymocytes at day 7 (not shown), medullary areas identified using the mTEC marker ERTR5, including Aire^+^ mTECs, were detectable after BMT, as were cortical areas identified by the cTEC marker CD205^+^ (Fig. S2). Thus, despite reduced mTEC frequency, the regenerating thymus showed organized cortex and medulla areas. Interestingly, while differences in cTECs and mTECs that occurred after BMT also occurred during age-related thymus atrophy (Venables et al., 2019), others (Ohigashi et al., 2015) reported full recovery of mTECs using 5.5-Gy sublethal irradiation, which contrasts with our results using 10-Gy lethal irradiation. Thus, responses of cTECs and mTECs to multiple forms of stimuli can differ, and further examination of this will likely aid in understanding their importance in relation to thymus function.

mTECs control the intrathymic DC pool that is important for negative selection and Foxp3^+^ Treg development (Cosway et al., 2018; Lei et al., 2011; Łyszkiewicz et al., 2015). To see if failed mTEC regeneration is accompanied by an impact on donor-derived thymic DC development after BMT, we subdivided thymic DCs (Fig. 1 E) into PDCA1^+^ plasmacytoid DC and conventional DC Sirpα^−^ (cDC1) and Sirpα^+^ (cDC2) subsets as described (Cosway et al., 2017). At 7 d after transplant, CD45.1^+^ donor-derived thymic DCs were undetectable (Fig. 1 E). Importantly, by 14 d after transplant, all three thymic DC subsets of donor origin had emerged simultaneously. However, their numbers were significantly reduced and remained low (Fig. 1 E), including at 28 d when donor-derived DCs were present at normal numbers in the spleen (not shown). Interestingly, 56 d after BMT, while total thymic DCs remained reduced, we found this defect was selectively within cDC1 (Fig. 1 E). Collectively, our findings show BMT in mice results in an imbalance in TEC microenvironments, characterized by depletion of mTECs but not cTECs. Furthermore, this is accompanied by a sustained failure in mTEC regeneration and inefficient recovery of the intrathymic DCs. As with mTECs, while the reasons for this differential recovery of thymic DC subsets is not clear, these mTEC/DC defects occur alongside unaltered cTEC frequencies, suggesting that while cortex aspects of thymus function after BMT remain largely intact, medulla-dependent processes may be impaired.

### Post-transplant recovery of conventional T cells occurs alongside impaired Foxp3^+^ Treg development

**Figure 2.**
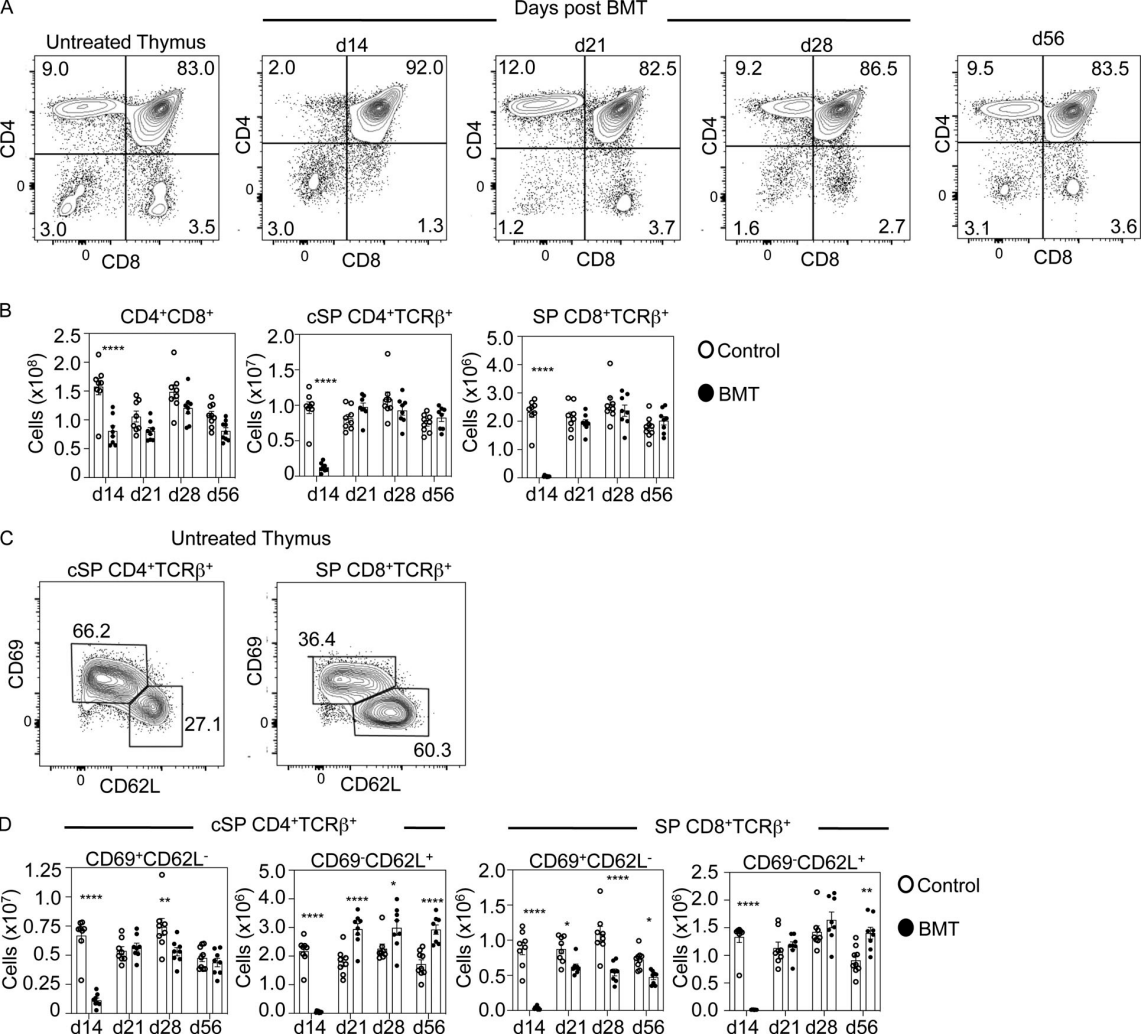
**Post-transplant thymopoietic recovery of conventional αβT cells occurs independently of thymus medulla regeneration.** **(A)** CD4/CD8 profiles of thymocytes from control or BMT mice at the indicated time points, the latter pregated on donor CD45.1^+^ cells. d, day. **(B)** Quantitation of CD4^+^CD8^+^ and conventional CD25^−^Foxp3^−^ (cSP) CD4^+^TCRβ^+^/CD8^+^TCRβ^+^ thymocytes at indicated time points in control (white dots) and BMT mice (black dots). **(C and D)** Subdivision of cSP CD4^+^TCRβ^+^ thymocytes into immature CD69^+^CD62L^−^ and mature CD69^−^CD62L^+^ subsets; quantitation of control (white dots) and BMT mice (black dots) is in D. All data from three separate experiments; *n* = 8 for each time point. Error bars indicate SEM; *, P < 0.05; **, P < 0.01; ****, P < 0.0001.

To relate mTEC changes to functional abilities of the post-transplant thymus, we analyzed development of donor-derived conventional αβT cells and Foxp3^+^ Tregs. While donor-derived CD4^+^CD8^+^ thymocytes were completely absent at 7 d (not shown), a large cohort was readily detectable from day 14 until day 56 (Fig. 2, A and B). Moreover, while mature conventional SP (cSP) CD4^+^TCRβ^+^CD25^−^Foxp3^−^ and SP CD8^+^TCRβ^+^ thymocytes were almost absent at 14 d, their recovery was complete by 21 d, including successful recovery of mature CD69^−^CD62L^+^ cells (Fig. 2, B–D). This recovery of cSP thymocytes was maintained until at least 56 d after transplant (Fig. 2, B and D). Thus, in the absence of an intact mTEC compartment, the post-transplant thymus supports effective restoration of conventional αβT cell development from donor progenitors. Interestingly, this “medullary independence” of conventional αβT cells during immune reconstitution mirrors events in the steady-state thymus (Cowan et al., 2013), suggesting that conventional T cell development is controlled similarly in both steady-state and regenerating thymic microenvironments.

**Figure 3.**
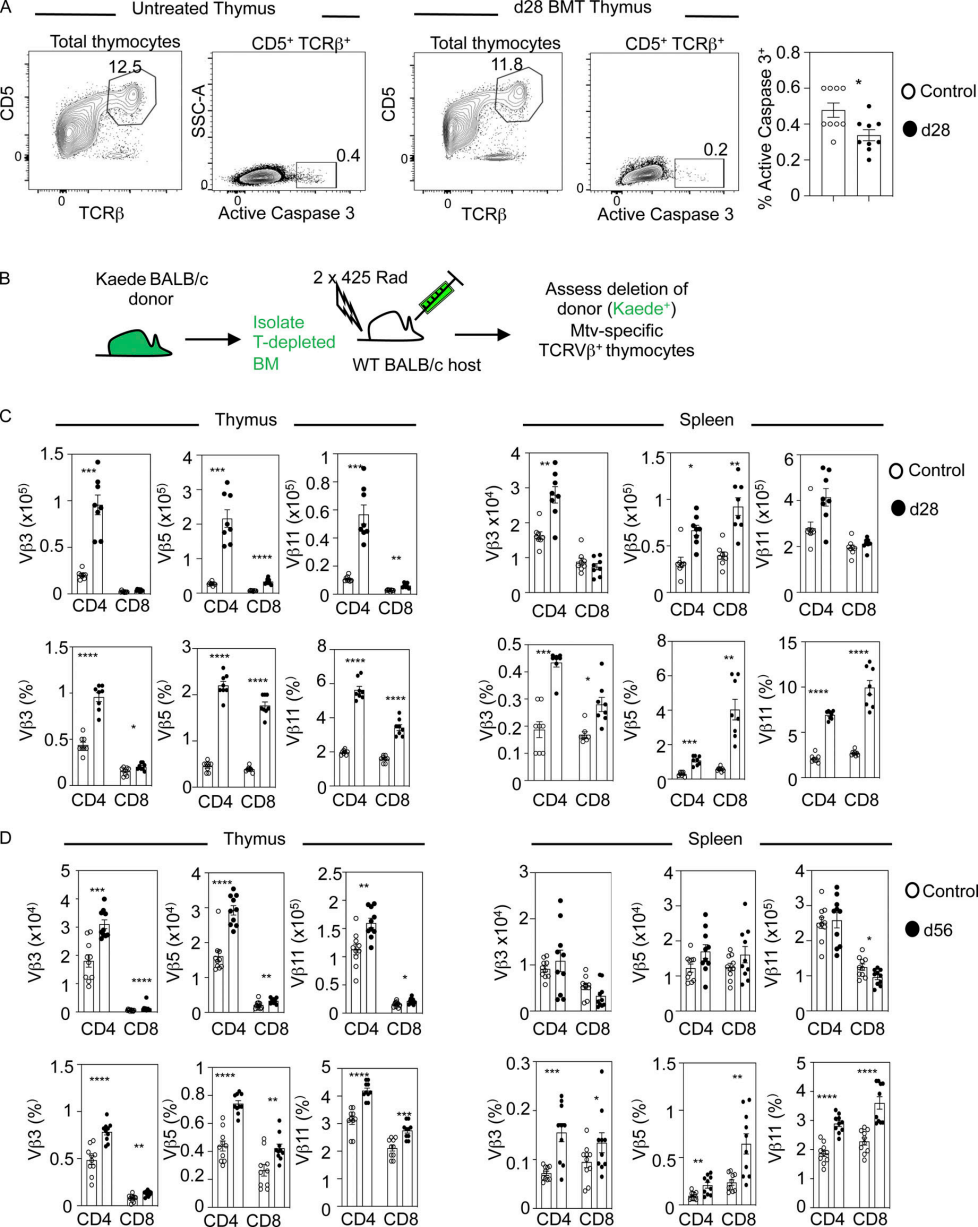
**Immune reconstitution involves defective negative selection of conventional αβT cells.** **(A)** Detection of TCR-signaled cleaved caspase 3^+^ thymocytes undergoing negative selection in control (white dots) or in BMT mice at 28 d (d28) after transplant (black dots). Data are from three separate experiments; *n* = 9 for each time point. **(B)** Schematic for generation of BALB/c background BM chimeras, where expression of the green fluorescent protein Kaede enables identification of donor-derived cells. **(C and D)** Quantitation of TCRVβ3^+^, Vβ5^+^, and Vβ11^+^ SP CD4^+^TCRβ^+^ or SP CD8^+^TCRβ^+^ in thymus (left panel) or spleen (right panel) with control (white dots) or BMT mice at 28 d after transplant (black dots; C) or 56 d after transplant (D). For BMT mice in C and D, cells were pregated on Kaede^+^ cells of donor origin. Data from three separate experiments; *n* = 8–10 per time point. Error bars indicate SEM. *, P < 0.05; **, P < 0.01; ***, P < 0.001; ****, P < 0.0001. SSC-A, side scatter area.

**Figure S3.**
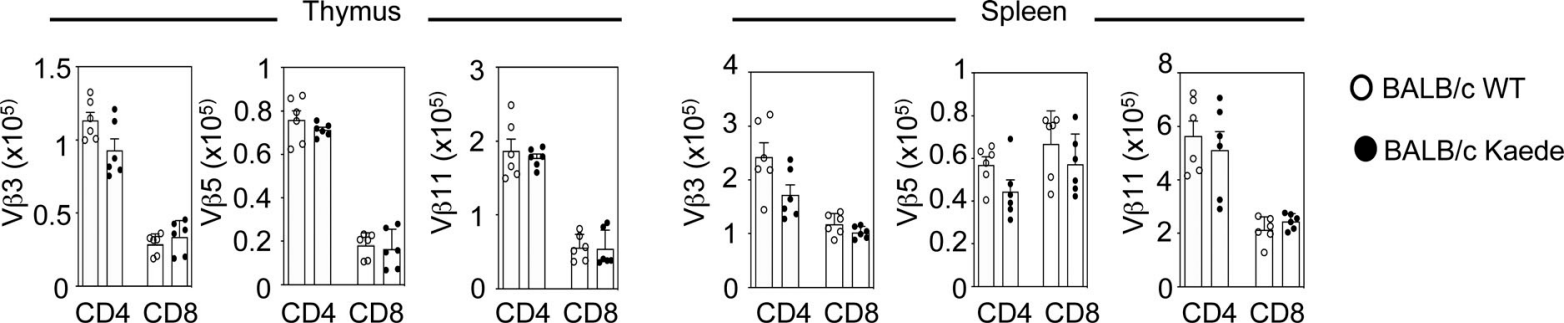
**Comparable TCRVβ usage in BALB/c and BALB/c Kaede mice.** Analysis of TCRVβ3^+^, 5^+^, and 11^+^ CD4^+^ and CD8^+^ SP cells in thymus and spleen of WT BALB/c and Kaede BALB/c mice (minimum of six mice from two separate experiments). Error bars indicate SEM.

While the presence of SP thymocytes indicates recovery of donor-derived thymopoiesis, it does not inform on the ability of the thymus to impose tolerance. To address this, we analyzed negative selection in the polyclonal αβTCR repertoire via detection of cleaved caspase 3 (Breed et al., 2019). As described previously (Breed et al., 2019), we used CD5 and TCRβ to look within bulk thymocytes undergoing intrathymic selection irrespective of their CD4/CD8 phenotype. When thymocytes were harvested 28 d after transplant, compared with untreated control mice we saw a significant reduction in TCRβ^hi^CD5^hi^ cleaved caspase 3^+^ cells, suggesting fewer thymocytes were undergoing negative selection (Fig. 3 A). To examine this further, we studied deletion of Vβ3^+^, Vβ5^+^, and Vβ11^+^ thymocytes in mammary tumor virus (Mtv)–expressing BALB/c mice. Here, Mtv superantigens carried by BALB/c mice cause intrathymic deletion of specific Vβ subsets, with an increased frequency of SP thymocytes and T cells expressing these specific V-gene families indicating defective negative selection (Dyson et al., 1991; Frankel et al., 1991; Lei et al., 2011; Moore et al., 1994). We used flow cytometric analysis and antibodies to specific TCRVβ chains to analyze negative selection with this approach. Also, as analysis of Mtv-mediated negative selection requires BALB/c mice, and congenic CD45.1/CD45.1 strains were not available to us on this background, we transplanted T-depleted BM from BALB/c Kaede donors into lethally irradiated WT BALB/c hosts (Fig. 3 B). Here, we were able to readily identify donor-derived cells on the basis of their expression of the green fluorescent protein Kaede (Mackley et al., 2015; Tomura et al., 2008). Importantly, relative usage of TCRVβ chains was comparable in WT BALB/c and Kaede BALB/c mice (Fig. S3). When chimeras were harvested 28 d after transplant, and in contrast to unmanipulated BALB/c mice, we found that intrathymic deletion of CD4^+^ and CD8^+^ Vβ3^+^, Vβ5^+^, and Vβ11^+^ thymocytes was impaired (Fig. 3 C). Importantly, increased percentages of Vβ3^+^, Vβ5^+^, and Vβ11^+^ CD4^+^ and CD8^+^ αβT cells were also detectable in the periphery of BM chimeras (Fig. 3 C), alongside increased numbers of Vβ3 (CD4^+^) and Vβ5 (CD4^+^ and CD8^+^), indicating that self-reactive T cells can leave the thymus after BMT. Interestingly, at 56 d after BMT, Vβ3, Vβ5, and Vβ11 CD4^+^ and CD8^+^ cells were increased in both number and percentage in the thymus, and were increased in percentage but not number in the spleen (Fig. 3 D). Thus, analysis of Mtv-mediated negative selection demonstrates an inefficiency in this process that correlates with thymus medulla defects and, at least at 28 d after BMT, results in the escape of Mtv-reactive αβT cells into peripheral tissues. Interestingly, in other studies, effective negative selection using either allogeneic BMT to assess Mtv-mediated deletion (Marrack et al., 1988; Speiser et al., 1989) or TCR transgenic mice to study antigen-specific deletion (Hubert et al., 2011; Liston et al., 2003) has been reported. While the reasons for this difference to our study are not fully clear, they may reflect differences in experimental approaches used (e.g., TCR transgenic versus non–TCR transgenic); it is also important to emphasize that unlike previous Mtv-based studies, our study involves analysis of Mtv-mediated deletion in a syngeneic BMT transplant system.

**Figure 4.**
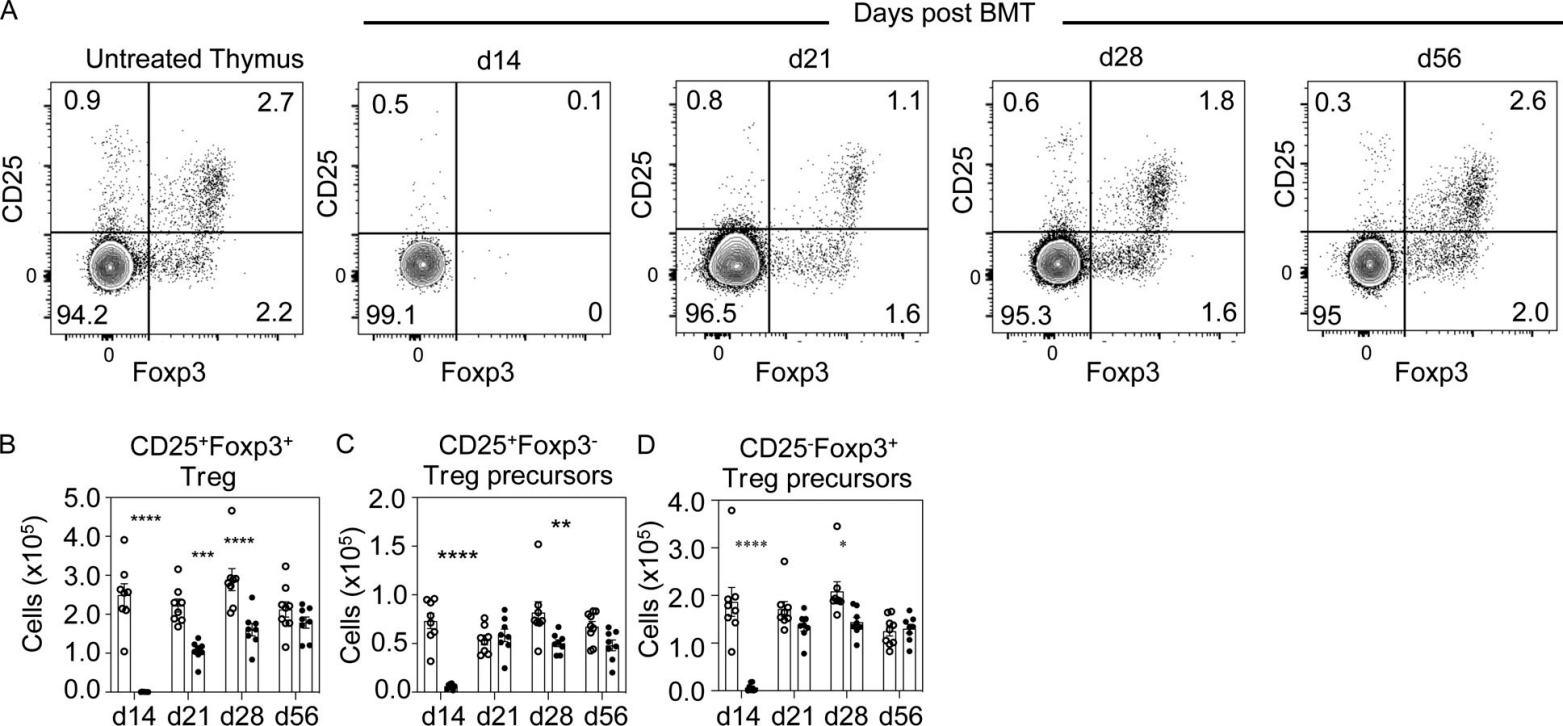
**Foxp3^+^ Treg development is impaired during post-transplant immune reconstitution.** **(A)** Representative FACS plots of SP CD4^+^TCRβ^+^ thymocytes in untreated and BMT mice analyzed for expression of Foxp3 and CD25, with thymocytes from BMT mice pregated on donor-derived CD45.1^+^ cells. d, day. **(B–D)** Quantitation of SP CD4^+^TCRβ^+^CD25^+^Foxp3^+^ Treg (B), SP CD4^+^TCRβ^+^CD25^+^Foxp3^−^ Treg precursors (C), and SP CD4^+^TCRβ^+^CD25^−^Foxp3^+^ Treg (D); white dots denote control mice, and black dots show BMT mice harvested at the indicated time points. Data from three separate experiments; *n* = 8 at each time point. Error bars indicate SEM. *, P < 0.05; **, P < 0.01; ***, P < 0.001; ****, P < 0.0001.

Alongside negative selection, Foxp3^+^ Treg production is essential for immune tolerance. Consistent with the absence of cSP CD4^+^ thymocytes at 14 d (Fig. 2, B–D), SP CD4^+^CD25^+^Foxp3^+^ Treg and CD25^−^Foxp3^+^/CD25^+^Foxp3^−^ Treg precursors (Lio and Hsieh, 2008; Tai et al., 2013) were also absent at this time point (Fig. 4, A and B). However, we saw significantly reduced CD4^+^CD25^+^Foxp3^+^ Treg numbers until 28 d, a stage when cSP CD4^+^ thymocytes were restored to normal frequency (Fig. 2 B), with no difference in Treg numbers between control and BMT mice by day 56. Interestingly, apart from a reduction in CD25^+^Foxp3^−^ cells at 28 d after transplant, Treg precursors remained largely unchanged between 21 d and 56 d in BMT and control mice (Fig. 4, C and D). While the reasons for this differential recovery is not known, our observations suggest an uncoupling of Treg precursors and their mature Treg progeny. Here, it is interesting to note that both Treg and CD25^+^ Treg precursors can respond to IL-2 (Lio and Hsieh, 2008), indicating that reduced intrathymic availability of IL-2 and/or Treg/progenitor competition for IL-2 may be one factor in explaining this kinetic of Treg development in the post-BMT thymus. Collectively, our data show that defects in post-transplant thymus medulla recovery are mirrored by limitations in Foxp3^+^ Treg development, which fits well with the medulla dependency of these cells (Cowan et al., 2013; Perry et al., 2014). Together, these findings suggest the post-transplant thymus supports generation of conventional αβT cells before reestablishment of the key intrathymic tolerance mechanisms of negative selection and Foxp3^+^ Treg development.

### Defective medulla regeneration allows escape from negative selection and loss of post-transplant tolerance

**Figure 5.**
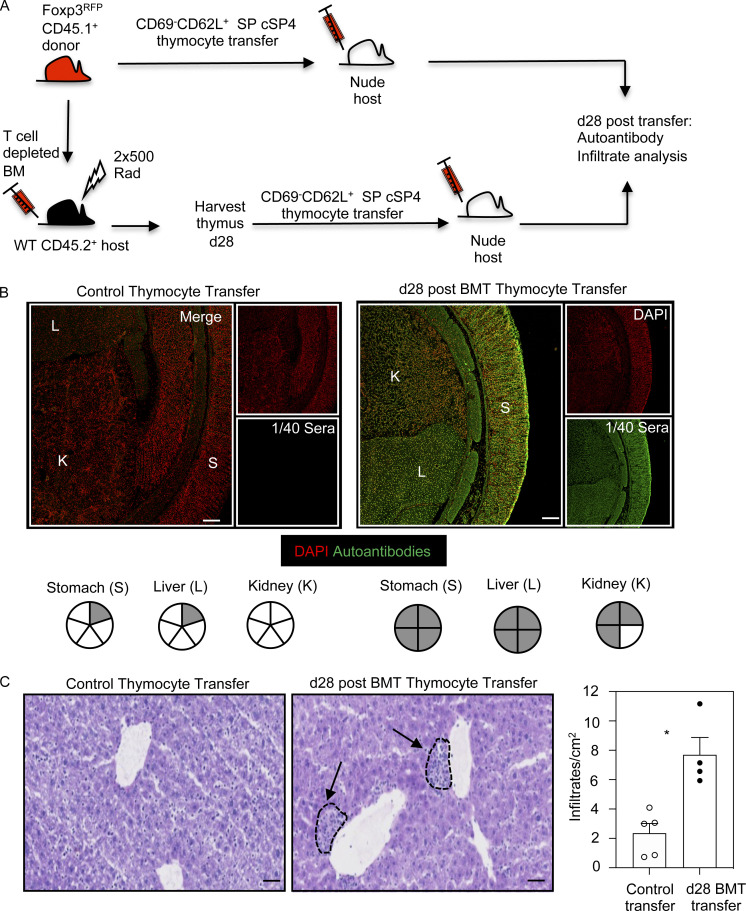
**Failures in post-transplant thymus medulla generation result in loss of tolerance and autoimmunity.** **(A)** Schematic of the generation of BMT mice to study induction of T cell tolerance during immune reconstitution. **(B)** Confocal images of kidney (K), liver (L), and stomach (S) sections incubated with 1/40 sera obtained from nude mice receiving mature cSP CD4^+^ thymocytes from either control mice or day 28 (d28) BMT mice. Autoantibody staining is shown in green, and DAPI is in red. Scale bar denotes 100 µm. Images represent serum staining from at least four mice and three separate sections per mouse. Pie charts summarize autoantibody staining data; each segment represents an individual mouse, and gray denotes positive autoantibody staining. **(C)** Liver sections from control or day 28 BMT mice, stained with hematoxylin and eosin to detect lymphocytic infiltrates (indicated by dotted lines and arrows). Scale bars denote 50 µm. Bar chart shows quantitation of infiltrates in control (white dots) and day 28 BMT mice (black dots). Data from four or five mice and two separate experiments. Error bars indicate SEM; *, P < 0.05.

In the steady state, defective thymus medulla development causes tolerance breakdown and autoimmunity (Boehm et al., 2003; Cosway et al., 2017; Weih et al., 1995). In BMT mice, analysis of immune tolerance in relation to intrathymic mechanisms is made difficult because of the need for effective reconstitution of additional immune populations such as B cells and DCs, which are necessary to drive self-reactive T cell immunity. To circumvent this problem, we set up experiments to see whether SP thymocytes generated during BMT would initiate autoimmune symptoms in unmanipulated athymic nude mice, where B cells and DCs are present. Thus, initial BM chimeras were made using C57BL/6 CD45.1^+^Foxp3^RFP^ BM donors and lethally irradiated CD45.2^+^ C57BL/6 hosts. To test the tolerance state of cSP4 thymocytes after BMT, mature CD45.1^+^ SP CD4^+^CD69^−^CD62L^+^ thymocytes that lacked Foxp3^RFP^ expression were then sorted from either 28-d BM chimeras or unmanipulated CD45.1^+^Foxp3^RFP^ control mice (Fig. 5 A). To assess any tolerance deficiencies in the T cell compartment, sorted thymocytes were i.v. injected into unmanipulated nude hosts, and tissues and sera were taken for analysis after a further 28 d after cell transfer. Strikingly, all (4/4) nude hosts receiving SP CD4^+^ thymocytes from BMT mice contained autoantibodies to stomach and liver, with 3/4 also showing autoantibodies to kidney (Fig. 5 B). This was in marked contrast to the paucity of autoantibodies (0/5 kidney, 1/5 stomach, 1/5 liver) in nude mice receiving SP CD4^+^ thymocytes from control mice (Fig. 5 B). Moreover, significantly more lymphocytic infiltrates were detectable in mice receiving thymocytes from BMT mice compared with control thymocytes (Fig. 5 C). Thus, immune reconstitution following syngeneic BMT results in autoimmunity caused by failures in thymus medulla regeneration.

Immune reconstitution via BMT is important to clinically restore T cell immunity. The thymus plays an essential role in this process by fostering T cell development from donor-derived progenitors (Awong et al., 2013; Hakim et al., 2005; Singh et al., 2020). In addition, the thymus must ensure that thymopoiesis is synchronized with tolerance mechanisms to regenerate immunologically “safe” and self-tolerant T cells. Here, we report failures in thymus medulla regeneration, which cause a functional uncoupling of T cell development and tolerance, resulting in the escape of self-reactive T cells and autoimmunity. These findings have several important implications. First, from a technical perspective, the existence of long-lasting defects in thymic microenvironments in mouse BM chimeras is relevant to their widespread use as an experimental model to study the immune system (Arber et al., 2013). Indeed, we demonstrate an incomplete recovery of thymus function in this model, where the medullary-dependent events required to impose T cell tolerance are lacking. Thus, experiments involving BMT should be interpreted with caution, particularly in relation to study of the medulla and thymic tolerance.

Second, our study may also be relevant to the use of BMT as a clinical therapy. Here, it is interesting that a side effect of allogeneic BMT is GVHD, caused by induction of host-reactive T cell responses (Arber et al., 2013; Barton-Burke et al., 2008; Cooke et al., 2017). We used autologous BMT, meaning signs of autoimmunity shown here are not caused by allogeneic responses. Importantly however, cases of GVHD termed “auto-GVHD” have been reported in an autologous BMT setting (El-Jurdi et al., 2017; Kline et al., 2008). Similar to our experiments using syngeneic host and donor mice, such symptoms cannot be explained by allogeneic T cell responses. As GVHD and autoimmunity can share common clinical features, this raises the possibility that some GVHD cases are manifestations of self-reactivity that may be explained by failures in thymus medulla function and loss of thymic tolerance. While links between BMT and autoimmunity require further investigation, if this is the case, then post-transplant analysis of medulla-dependent functions, including Foxp3^+^ Treg development, may be a means to measure the success or failure of thymus medulla reestablishment and monitoring of autoimmune susceptibility. Here, it is interesting to compare thymus recovery at 28 d and 56 d after BMT. At 28 d, mTEC deficiencies occurred alongside defects in thymic DCs, Treg development, negative selection, and tolerance. At 56 d, defects in mTECs, thymic DCs, and negative selection persisted, while Treg development was restored. Importantly, it is unclear whether loss of tolerance shown here at 28 d after transplant was caused by individual defects in negative selection or Treg generation, or a combination of both. Indeed, analysis of “forbidden” TCRVβ-expressing cells showed increased numbers in the periphery at 28 d but not 56 d after BMT. This may be consistent with the idea that while delayed mTEC recovery leads to reduced negative selection of conventional thymocytes, this is either mitigated in the periphery by the restoration of Tregs, or that some of these self-reactive cells are unable to escape the thymus.

Relevant to this, it is also unclear how defects in negative selection shown here compare with other studies. However, it is interesting to note that analysis of Mtv-mediated deletion in mice lacking expression of the Aire-dependent chemokine XCL1 showed increased frequencies of Vβ3, Vβ5, and Vβ11 SP4 thymocytes similar to that shown here, with XCL-1–deficient mice (Lei et al., 2011) also showing features of autoimmunity similar to those presented here. Also relevant are studies indicating that effective representation of tissue-restricted antigens (TRAs) can be achieved by small numbers of mTECs (Breed et al., 2018). Thus, while the mTEC decline seen after BMT may not impact intrathymic TRA representation, the paucity of DCs may limit cross-presentation of TRA-derived peptides, which contributes to thymic tolerance mechanisms (Breed et al., 2018). Further work is needed to examine the impact of specific medulla deficiencies on thymic tolerance after BMT.

It is also interesting that defects in TEC generation after BMT are restricted to mTECs. Indeed, effective generation and positive selection of CD4^+^CD8^+^ thymocytes demonstrates that cTEC functions remain intact during immune reconstitution. Why these selective failures in mTEC regeneration take place is unclear. Given that SP thymocytes generated during immune reconstitution are correctly positioned within medullary areas (not shown), this is unlikely to be due to a lack of SP thymocyte/mTEC crosstalk (Hikosaka et al., 2008; Irla et al., 2008; Rossi et al., 2007). While earlier studies showed mTECs may be more susceptible than cTECs to therapeutic interventions associated with transplantation, including preconditioning regimens (Fletcher et al., 2009; Kanariou et al., 1989), it is important to note that the impact of such treatments on central tolerance were not addressed by these studies.

An alternative explanation for failed medulla regeneration is progressive loss of mTEC progenitors that occurs during steady-state thymopoiesis (Hamazaki et al., 2016; Sekai et al., 2014). This erosion of progenitor availability in adulthood may then leave the medulla unable to regenerate effectively following ablative therapy. Importantly, such experiments focused on development of Aire^+^ mTECs produced from RANK^+^ progenitors (Hikosaka et al., 2008; Rossi et al., 2007), and attempts to manipulate the medulla often focus on RANK-mediated pathways (Khan et al., 2014; Lopes et al., 2017). Importantly, it is not known whether RANK^+^ progenitors also give rise to other mTEC subsets, including CCL21^+^ mTEC^lo^ (James et al., 2021; Kozai et al., 2017). If dedicated progenitors exist for functionally distinct mature mTEC subsets, medulla regeneration after BMT may be skewed in a manner that is controlled by distinct mTEC progenitor availability. Further experiments are needed to identify adult mTEC progenitors, which should aid approaches to improve medulla recovery.

In sum, during BMT-dependent immune reconstitution, we examined the ability of the thymus to support thymopoiesis and T cell tolerance, two important processes that are highly coordinated in the steady-state thymus. We found that recovery of donor-derived thymopoiesis in the post-transplant thymus precedes reestablishment of thymic tolerance mechanisms, which occurs alongside failures in medulla regeneration and escape of self-reactive T cells. These findings demonstrate the importance of the medulla in ensuring balanced production of αβT cells in the thymus in a therapeutic setting. They also highlight the importance of protecting/boosting medulla development alongside BMT to improve the treatment of immune disorders that require effective and safe T cell reconstitution.

## Materials and methods

### Mice

C57BL/6 (CD45.2^+^) or BALB/c (CD45.2^+^) mice, aged between 6 and 8 wk at the start of experiments, were used as BMT hosts as indicated. Female mice were used throughout the study for both hosts and donors. Mice for BMT and controls were purchased from Charles River. For BM donors, WT BoyJ (CD45.1^+^) or Kaede BALB/c mice aged between 6 and 8 wk were used. CD45.1^+^Foxp3^RFP^ (Wan and Flavell, 2005) mice (6–8 wk old) were used in BMT experiments involving thymocyte transfers into 10-wk-old C57BL/6 nude hosts. All strains were housed within the Biomedical Services Unit at the University of Birmingham. Approval for all experiments was obtained from the Birmingham Animal Welfare and Ethical Review Body and the UK Home Office.

### Generation of BM chimeras

BM chimeras were generated using standard techniques. In brief, host mice were lethally irradiated (for C57BL/6 mice: split dose of 2 × 500 rad; for BALB/c mice: split dose of 2 × 425 rad; Dudakov et al., 2012; Lucas et al., 2016). For experiments involving C57BL/6 hosts, either congenic CD45.1^+^ or CD45.1^+^Foxp3^RFP^ donor BM was used, while for BALB/c hosts, we used BALB/c Kaede^+^ BM. In all cases, BM was flushed from the tibias and femurs of donor mice and depleted of T cells using anti-CD3 phycoerythrin (PE) and anti-PE microbeads (Miltenyi Biotec). For immune reconstitution, 5 × 10^6^ T-depleted BM cells were then injected into the tail vein of irradiated mice, which were harvested at the indicated time point. A cohort of age-matched unmanipulated mice was allocated alongside each set of chimeras to act as controls at the point of harvest.

### Antibodies and cell sorting

For TEC analysis (Lucas et al., 2020), thymus tissue was enzymatically digested using Collagenase/Dispase (Roche) and DNase I (Sigma-Aldrich). Reagents used (eBioscience unless otherwise stated) are anti-EpCAM1 PerCP-Cy5.5 (G8.8), anti-CD45 APC-Cy7 (30-F11), anti-Ly51 PE (6C3), anti-MHCII Pacific Blue (M5/114.15.2), anti-Aire Alexa Fluor 488 (5H12), CD80 BV605 (16-10A1; BioLegend), and UEA-1 biotin (Vector Laboratories) detected with streptavidin PE-Cy7. For thymic DC analysis, Collagenase D– and DNase I–digested thymus samples were stained with a lineage cocktail of FITC-labeled antibodies: anti-CD3 (145-2C11), anti-CD19 (eBio1D3), anti-NK1.1 (PK136) alongside anti–PDCA-1 Pacific Blue (129C1; BioLegend), anti-CD11c PE-Cy7 (N418), and anti-Sirpα PE (P84). For thymocyte analysis, thymus tissue was teased apart between glass slides and stained with the following: anti-CD45.1 PECy7 (A20), anti-CD4 BV711 (RM4-5; BioLegend), anti-CD8 BV786 (53–6.7; BioLegend), anti-TCRβ APC-Cy7 (H57-597), anti-CD25 BV650 (PC61.5), anti-CD5 Biotin (53–7.3) detected with streptavidin PE-Cy7, anti-CD69 PerCP-Cy5.5 (H1.2F3), anti-CD62L APC (MEL-14), and anti-Foxp3 PE (FJK-165). For analysis of negative selection, thymocytes were stained as described (Breed et al., 2019) using a lineage cocktail of Pacific Blue–labeled antibodies (anti-CD19, anti-NK1.1, and anti-CD25), anti-TCRδ (GL3) together with anti-CD5 biotin (53–7.3) detected with streptavidin PECy7, anti-TCRβ APC-Cy7 (H57-597), and anti-cleaved Caspase-3 PE (Asp175; 5AIE; Cell Signaling Technologies). Intracellular staining for negative selection analysis was performed using a BD Cytofix/Cytoperm kit.

### Analysis of autoimmunity after BMT

For cell transfer experiments, CD45.1^+^ SP CD4^+^CD69^−^CD62L^+^Foxp3^RFP−^ conventional mature thymocytes were sorted from either unmanipulated or day 28 BM chimera mice using a BD FACS Aria, to a purity >99% (not shown). Sorted cell populations were separately injected into the tail veins of nude mice (10^6^ cells per mouse), which were then harvested a further 28 d later. To detect autoantibodies (Cosway et al., 2017), composite tissue slides (INOVA Diagnostics) containing rat liver, kidney, and stomach were used that were preblocked with 10% goat serum (Sigma-Aldrich). Serum samples at 1/40 dilution were added to slides, which were detected with goat F(ab′)_2_ anti-mouse IgG(H+L) FITC (Southern Biotech). After antibody labeling, slides were dipped in DAPI, washed in PBS, and analyzed using a Zen 880 microscope and Zen Black software (Zeiss). Autoantibody staining was assessed by two independent researchers, using an arbitrary scale of 1–6 based on staining intensity. Lymphocytic infiltrates were examined exactly as described (Cosway et al., 2017). Briefly, liver tissue from cell-transferred nude mice was first embedded in optimal cutting temperature compound (Sakura Finetek), then snap-frozen in liquid nitrogen. 7-µm acetone-fixed sections were then stained with hematoxylin and eosin and imaged using an Axio ScanZ1 microscope (Zeiss). Between three and five sections were used for quantitation, performed by counting individually discrete cellular accumulations per tissue at 30–40 µm apart, with infiltrates identified as >25 cells clustered together.

### Analysis of Mtv-mediated negative selection

Thymus and spleen tissue from control BALB/c mice and BALB/c-BALB/c Kaede chimeras was mechanically disrupted and stained with anti-CD4 BV711 (RM4-5; BioLegend), anti-CD8 BV786 (53–6.7; BioLegend), anti-TCRβ APC-Cy7 (H57-597; eBioscience), and PE-conjugated antibodies (all BD Biosciences) to TCRVβ3 (KJ25), TCRVβ5 (MR9-4), and TCRVβ11 (RR3-15). Data on percentages and numbers of TCRVβ^+^ cells from indicated tissues are shown after pregating on SP CD4^+^TCRβ^+^ cells or SP CD8^+^TCRβ^+^ cells as indicated.

### Confocal microscopy

Thymus tissues from control and BMT mice were snap-frozen and mounted in OCT, and 7-µm sections were cut and fixed in acetone (Cosway et al., 2017). Sections were stained with the following (eBioscience unless stated otherwise): anti-Aire Alexa Fluor 488 (4H12), anti-CD205 biotin (205yetka) detected with streptavidin Alexa Fluor 555 (Thermo Fisher Scientific), ERTR5 (gift from W. van Ewijk; Leiden University Medical Centre, Leiden, Netherlands) detected with goat anti-rat IgM Alexa Fluor 647 (Thermo Fisher Scientific). Labeled slides were dipped in DAPI (Sigma-Aldrich), covered with Prolong Gold (Thermo Fisher Scientific) before coverslip mounting, and imaged using a Zeiss LSM 880 microscope and Zen black software.

### Statistical analysis

All statistical analyses were performed using GraphPad Prism 9.1. For comparison between age-matched controls and BMT mice, an unpaired Student’s *t* test was used. Only P values below 0.05 were noted as significant, as follows: *, P < 0.05; **, P < 0.01; ***, P < 0.001; ****, P < 0.0001; any nonsignificant differences were not specified. All error bars represent SEM.

### Online supplemental material

Fig. S1 shows total thymus cellularity across all time points analyzed after BMT, cTEC/mTEC gating, and number of CD4^+^ and CD8^+^ αβT cells in the spleen of age-matched control and BMT mice (donor-derived CD45.1^+^ cells for the latter) at the indicated time points after transplant (minimum of eight mice from at least three separate experiments). Fig. S2 shows confocal analysis of frozen thymus sections from untreated and post-BMT mice at the indicated time points, labeled with CD205 and ERTR5 to identify cortex and medulla and ERTR5 and Aire to detect Aire^+^ mTECs. Fig. S3 shows analysis of TCRVβ3^+^, 5^+^, and 11^+^ CD4^+^ and CD8^+^ SP cells in thymus and spleen of WT BALB/c and Kaede BALB/c mice (minimum of six mice from two separate experiments).
